# *PDGFD* maintains ovine tail ADSCs in a proliferative state by suppressing *CXCL8* and activating PI3K/MAPK signaling

**DOI:** 10.3389/fvets.2026.1777426

**Published:** 2026-02-13

**Authors:** Jilong Han, Qianqian Liang, Jie Zhang, Xianshu Wang, Beibit Turganbekovich Kulatayev, Mahaba Rouzi, Pengchen Wan, Min Yang

**Affiliations:** 1 College of Animal Science and Technology, Shihezi University, Shihezi, China; 2 Department of Zooengineering and Biotechnology, Kazakh National Agrarian Research University, Almaty, Kazakhstan; 3 Xinjiang Uygur Autonomous Regional Animal Husbandry Station, Urumqi, China; 4 State Key Laboratory of Sheep Genetic Improvement and Healthy Breeding, Institute of Animal Husbandry and Veterinary Sciences, Xinjiang Academy of Agricultural and Reclamation Sciences, Shihezi, China

**Keywords:** adipose tissue, ADSCs, CXCL8, PDGFD, PI3K/MAPK signaling

## Abstract

**Introduction:**

The fat tail in sheep is a distinctive adaptive trait, yet the cellular mechanisms controlling its development remain poorly understood. Although *PDGFD* is a high-priority candidate gene from selective sweep analyses, its precise cellular function and underlying molecular mechanisms are uncharacterized.

**Methods:**

We first defined E70–E80 as the critical fetal period for the initial formation of rump or tail adipose tissue in sheep. To delineate the functional role of *PDGFD*, we performed knockdown experiments in ovine adipose-derived stem cells (ADSCs) and conducted transcriptomic profiling.

**Results:**

*PDGFD* knockdown significantly inhibited ADSCs proliferation and concurrently upregulated key adipogenic markers (*PPARγ*, *FABP4*). Transcriptomics revealed that this phenotype was mediated primarily through the profound downregulation of the chemokine *CXCL8*. Pathway analysis demonstrated that the PDGFD-CXCL8 axis co-regulates adipose plasticity by modulating both the PI3K-Akt and MAPK signaling pathways.

**Conclusion:**

We conclude that *PDGFD* is a master regulator of adipose tissue plasticity, driving progenitor expansion via a CXCL8-dependent mechanism. This PDGFD-CXCL8 regulatory axis orchestrates the cellular groundwork essential for the extensive fat deposition that defines the ovine fat-tail phenotype.

## 1 Introduction

Fat-tailed and fat-rumped sheep, characteristic of semi-arid regions, are widely distributed across Eastern and Southern Africa, the Central Asian steppes, and the Middle East. In these breeds, the tail and rump adipose tissue serves as a specialized organ for dynamic energy storage and release, analogous to the camel’s hump, thereby enhancing tolerance to harsh environmental challenges such as food scarcity, cold, and drought (1). This remarkable adaptability is associated with the tissue’s strong heterogeneity and plasticity, which allows for rapid expansion and mobilization of lipid stores in response to seasonal fluctuations (2). However, the molecular mechanisms underpinning this plasticity, particularly those governing the dynamics of adipocyte precursors, remain largely unknown.

Adipose tissue is a highly plastic endocrine organ that plays a key role in maintaining energy metabolism in mammals. Its plasticity—the capacity to change in size and cellular composition—is influenced by genetics, anatomical distribution, and the proliferative and differentiation capacities of adipocyte precursors (3–6)The sheep tail/rump fat depot, shaped by natural selection for efficient energy buffering, therefore presents an exceptional *in vivo* model to investigate the mechanisms of adipose tissue plasticity underlying species-specific adaptation.

Genomic studies have consistently identified the *PDGFD* gene region as showing the strongest signatures of positive selection in fat-tailed breeds (7–10). The platelet-derived growth factor D (PDGFD) signals through PDGFRα and PDGFRβ to modulate adipose biology (11, 12). Intriguingly, PDGFD promotes the proliferation of adipocyte precursors but inhibits their differentiation (7, 9, 10, 13–15). This dual function, directly impacting the progenitor pool and fate commitment, positions PDGFD as a potential key regulator of adipose tissue plasticity. Given that plasticity fundamentally relies on the balance between precursor expansion and adipogenic differentiation, PDGFD emerges as a prime molecular candidate orchestrating this process in fat-tailed sheep. However, the precise intracellular signaling networks and downstream effectors mediating these effects in the context of ovine adipose development and adaptation are not defined.

Adipogenesis in adipose tissue is a cell process of differentiation from committed preadipocytes into mature adipocytes, and plays an important role in adipose development (16). Therefore, to mechanistically dissect the role of PDGFD in ovine adipose plasticity, we utilized primary adipose-derived stem cells (ADSCs) from fetal sheep tail-a model exhibiting robust self-renewal and adipogenic potential, thus ideal for dissecting cell-autonomous developmental mechanisms (17, 18). We performed functional knockdown of *PDGFD* to define its contribution to progenitor proliferation and adipogenic commitment. Integrated transcriptomic and pathway analyses then identified key downstream mediators, revealing a PDGFD-CXCL8 axis operating via the PI3K-Akt and MAPK pathways (19–21). Our findings establish PDGFD as a central regulator of adipose progenitor dynamics, providing a molecular framework for understanding the developmental basis of the fat-tail adaptation.

## 2 Materials and methods

### 2.1 Ovine fetus and tissue collection

Rump and tail adipose tissue was aseptically dissected from ovine fetuses (approximately 60–90 days of gestation, E60-E90) of Kazakh sheep (fat-rumped) and Hu sheep (fat-tailed) breeds (22, 23). Fetuses were collected from the Western Pastoral Slaughterhouse in Shihezi, Xinjiang. Specifically, adipose tissue from three Kazakh sheep fetuses at about E75 was for the primary isolation of adipose-derived stem cells (ADSCs) used in subsequent functional experiments. The collected tissues were immediately washed four times with phosphate-buffered saline (PBS) (Solarbio, Beijing, China) supplemented with 1% penicillin–streptomycin (Solarbio, Beijing, China). All animal experimental procedures were approved by the Biology Ethics Committee of Shihezi University (approval number is: A2020-34).

### 2.2 Isolation and primary culture of ADSCs

ADSCs were isolated as previously described (24), with the following critical modifications to optimize yield and viability. Briefly, the collected adipose tissues from the tail or rump were immediately transferred to a biosafety cabinet and placed into a new sterile Eppendorf tube. The tissue was washed three times with sterile phosphate-buffered saline (PBS) (Solarbio, Beijing, China) containing 1% penicillin/streptomycin to remove debris and red blood cells. Subsequently, it was minced into fine fragments (approximately 1 mm^3^ in size) using surgical scissors and digested with 0.5 mL of 0.25% trypsin for 3 min at room temperature. The digestion was terminated by adding twice the volume of complete culture medium. The digest was then filtered through a 100 μm cell strainer, and the filtrate was centrifuged at 1000 × *g* for 5 min. After discarding the supernatant, the pellet was resuspended in 1 mL of complete medium and plated into a 60 mm culture dish. Cells were incubated at 37 °C in a 5% CO₂ atmosphere. The medium was replaced after 24 h to remove non-adherent cells and debris, and subsequently every 2 days thereafter. Primary cells were passaged upon reaching 80% confluence. Cells from passages 5 (P5) were used for all subsequent experiments to ensure consistency and avoid senescence-related effects.

### 2.3 Cell passaging

For passaging, cells were washed once with PBS to remove residual serum. Pre-warmed 0.25% trypsin (TransGen Biotech, Beijing, China) was added to cover the cell layer and incubated at 37 °C for 90 s. Cell detachment was monitored under a microscope until most of the cells had rounded up and detached from the surface. The enzymatic reaction was promptly stopped by adding twice the volume of complete medium. The cell suspension was collected, centrifuged at 300 × *g* for 5 min, and the pellet was resuspended in fresh complete medium for reseeding.

### 2.4 Characterization of ADSCs

Cells at passages 5 (P5) were characterized to confirm their identity as mesenchymal stem cells, based on their adherence to plastic, multilineage differentiation potential, and surface marker expression profile. Multilineage Differentiation Potential: ADSCs were induced toward adipogenic and osteogenic lineages as detailed in section 2.5.

Immunofluorescence Staining for ADSCs Surface Marker Identification: To morphologically confirm the stem cell properties of the isolated cells, we employed immunofluorescence staining to detect the expression of the ADSCs surface marker CD44. Specifically, P5 ADSCs were seeded in a 24-well plate at an appropriate density and allowed to grow to 70% confluence before fixation and staining. Cells were first fixed with 4% paraformaldehyde (BioSharp, Hefei, China) at room temperature for 20 min, followed by permeabilization with 0.2% Triton X-100 (Sigma-Aldrich, Germany) for 10 min to increase membrane permeability. To reduce non-specific binding, cells were blocked with 1% bovine serum albumin (BSA, Bioss, Beijing, China) at room temperature for 30 min. After blocking, cells were incubated overnight at 4 °C with a primary antibody against CD44 (rabbit polyclonal antibody, Bioss, Cat No. bs-55039R, dilution 1:1,000). The next day, after thorough washing with PBS, cells were incubated with a CoraLite488-conjugated goat anti-rabbit IgG secondary antibody (Wuhan Sanying, Cat No. SA00013-2, dilution 1:100) at room temperature in the dark for 1 h. Finally, nuclei were counterstained with DAPI (Sigma-Aldrich, Germany) for 10 min. Following staining, images were captured using a BX43 fluorescent microscope (Olympus Corporation, Japan).

### 2.5 Multilineage differentiation and staining

Adipogenic Differentiation: P5 ADSCs were induced to differentiate when they reached 95% confluence. The adipogenic induction medium, consisting of 90% DMEM/F12, 10% FBS, 10 ng/mL insulin, 1 μM dexamethasone, 1 μM rosiglitazone, and 0.5 mM IBMX, was applied for 2 days. The medium was then replaced with an adipogenic maintenance medium (90% DMEM/F12, 10% FBS, 10 ng/mL insulin, and 1 μM rosiglitazone), which was refreshed every 2 days until day 6. On day 6, cells were fixed with 4% paraformaldehyde for 20 min and stained with a 0.4% Oil Red O solution (Beyotime, Beijing, China) for 1 h to visualize lipid droplets. The expression of adipogenic genes (*PPARr*, *INHBA*, *PLIN1* and *FABP4*). and *PDGFD* and its receptor genes were analyzed by RT-qPCR at days 0, 2, 4, and 6 of differentiation (Supplementary Tables S2 for primers).

Osteogenic Differentiation: P5 ADSCs were induced at 80% confluence. The growth medium was replaced with an osteogenic induction medium (growth medium supplemented with 100 ng/mL ascorbic acid, 20 mM *β*-glycerol phosphate, and 200 nM dexamethasone), which was refreshed every 2 days. On days 6 and 12 post-induction, cells were fixed with 4% paraformaldehyde and stained with a 0.2% Alizarin Red S solution (BBI Life Sciences Co., Ltd., Shenggong, Shanghai, China) for 1 h at room temperature in the dark to detect mineralized matrix deposition. Staining was visualized and imaged under a light microscope.

### 2.6 siRNA transfection and cell proliferation assay

Three small interfering RNAs (siRNAs) targeting *PDGFD* and a non-targeting scram-bled negative control (NC) siRNA were designed and synthesized by BBI Life Sciences (Shanghai, China). Sequences are listed in Supplementary Tables S3. The sheep P5 ADSCs, which were cultured, purified, and characterized from sheep embryos, were seeded on 12-well plates 1 day prior to transfection to reach 60%–70% confluence. Three PDGFD siRNAs and negative control siRNA (siNC) were transfected at a final concentration of 20 nM per well using EL Transfection Reagent (BBI Life Sciences). The knockdown efficiency of each siRNA was assessed by RT-qPCR as described in section 2.9.4.

For the cell proliferation assay, ADSCs were seeded in 96-well plates at a density of 2.0 × 10^4^ cells per well. The siRNA with the highest knockdown efficiency (si-PDGFD-1427) was selected to establish the *PDGFD* knockdown model in P5 ADSCs. Cells were transfected using a mixture containing the siRNA and transfection reagent in 10% Opti-MEM (BBI Life Sciences). Cell proliferation was then assessed using a Cell Counting Kit-8 (CCK-8; Beyotime, Beijing, China) according to the manufacturer’s instructions. Briefly, 10% CCK-8 solution was added to the culture medium, followed by incubation at 37 °C for 2 h. The optical density (OD) at 450 nm was measured for both the si-PDGFD-1427 and siNC groups using a microplate reader (Thermo Fisher, MA, United States) at 24, 48, and 72 h post-transfection. Each treatment group was assayed with five replicates.

### 2.7 RNA sequencing and Bioinformatic analysis

Total RNA was extracted from ADSCs transfected with si-PDGFD or NC siRNA for 24 and 48 h using Trizol (TransZol Up kit, TransGen Biotech, Beijing, China). Three independent biological replicates were performed for each group and time point. RNA quality was assessed using an Agilent 2100 Bioanalyzer (Agilent Technologies, Santa Clara, CA, United States), and only samples with an RNA Integrity Number (RIN) > 7.0 were used for library preparation. Sequencing libraries were constructed and sequenced on an Illumina platform by a commercial service provider. Raw sequencing reads were quality-filtered using FastQC (v0.11.5) to obtain clean data, which were then aligned to the sheep reference genome (GCF_000298735.2_Oar_v4.0_genomic) using HISAT2 (v2.0.4). Transcript assembly and quantification were performed with StringTie (v1.2.3). Differentially expressed genes (DEGs) were identified using the DESeq2 package (v1.38.3). A model matrix was designed with a single variable indicating the treatment group and si-PDGFD group. To control the false discovery rate (FDR) arising from multiple hypothesis testing, the *p*-values were adjusted using the Benjamini-Hochberg procedure. Genes with an adjusted *p*-value (FDR) < 0.05 and an absolute log_2_ fold change > 1 were considered statistically significant differentially expressed genes (DEGs).

### 2.8 Pathway and protein–protein interaction analysis

Gene Ontology (GO) and Kyoto Encyclopedia of Genes and Genomes (KEGG) pathway enrichment analyses (25) were performed on the differentially expressed genes (DEGs) using the clusterProfiler package in R. Additionally, Gene Set Enrichment Analysis (GSEA) was conducted on the full ranked gene list to identify KEGG pathways with subtle but coordinated expression changes (26). The ggplot2 package is then utilized to visualize the enrichment results. Based on the KEGG enrichment results, the PI3K-Akt and MAPK signaling pathways were selected for further analysis. A protein–protein interaction (PPI) network was constructed using the online STRING database^1^ for key DEGs involved in these pathways, including *CXCL8*.

### 2.9 RNA extraction, cDNA synthesis, and gene expression analysis

#### 2.9.1 RNA extraction and cDNA synthesis

Total RNA was extracted from ADSCs using the TransZol Up kit (TransGen Biotech, Beijing, China) according to the manufacturer’s instructions. RNA concentration and purity were assessed using a Nanodrop 2000c spectrophotometer (Thermo Fisher Scientific, United States). cDNA was synthesized from 2.5 μg of total RNA using the EasyScript® One-step gDNA Removal and cDNA Synthesis SuperMix (TransGen Biotech, Beijing, China) in a 20 μL reaction volume(42 °C for 15 min and 85 °C for 5 s).

#### 2.9.2 Quantitative real-time PCR (RT-qPCR)

Gene expression was analyzed by RT-qPCR using the ArtiCanCEO SYBR qPCR Mix (Tsingke, Beijing, China) on a LightCycler 96 system (Roche, Basel, Switzerland). The stability of the reference gene GAPDH was validated across all experimental conditions prior to analysis (CV < 0.5). Gene-specific primers (sequences in Supplementary Tables S1) were designed using NCBI Primer-Blast. Each reaction was performed in triplicate. Gene expression was normalized to GAPDH, and relative expression was calculated using the 2^−ΔΔCt^ method.

#### 2.9.3 Validation of RNA-Seq results by RT-qPCR

To validate the transcriptome sequencing results, the expression levels of several key differentially expressed geneswere quantified by RT-qPCR. This analysis was performed using the same RNA samples (*n* = 3 biological replicates per group) from the 24-h and 48-h post-transfection time points, following the methods described in sections 2.9.1 and 2.9.2 (primers listed in Supplementary Table S2).

#### 2.9.4 Assessment of siRNA knockdown efficiency by RT-qPCR

To assess the knockdown efficiency of PDGFD-targeting siRNAs, RNA extraction and RT-qPCR were performed as described in sections 2.9.1 and 2.9.2. Briefly, 24 or 48 h after siRNA transfection (as detailed in section 2.6), cells were washed with PBS, and total RNA was extracted using TransZol Up reagent (TransGen Biotech). cDNA was synthesized, and the relative expression of PDGFD was measured by RT-qPCR using gene-specific primers (Supplementary Tables S3). Knockdown efficiency was calculated relative to cells transfected with the negative control siRNA (siNC).

### 2.10 Statistical analysis

The specific statistical tests used are detailed in the respective figure legends. Pairwise comparisons between two groups were analyzed using an unpaired two-tailed Student’s *t*-test. Comparisons among three or more groups were analyzed using one-way ANOVA, followed by Tukey’s *post-hoc* test for multiple comparisons. Sample sizes (*n*) for each experiment, representing biological replicates, are indicated in the figure legends.

## 3 Results

### 3.1 Identification of critical developmental windows and dynamic expression of PDGFD signaling component

**Figure 1:**
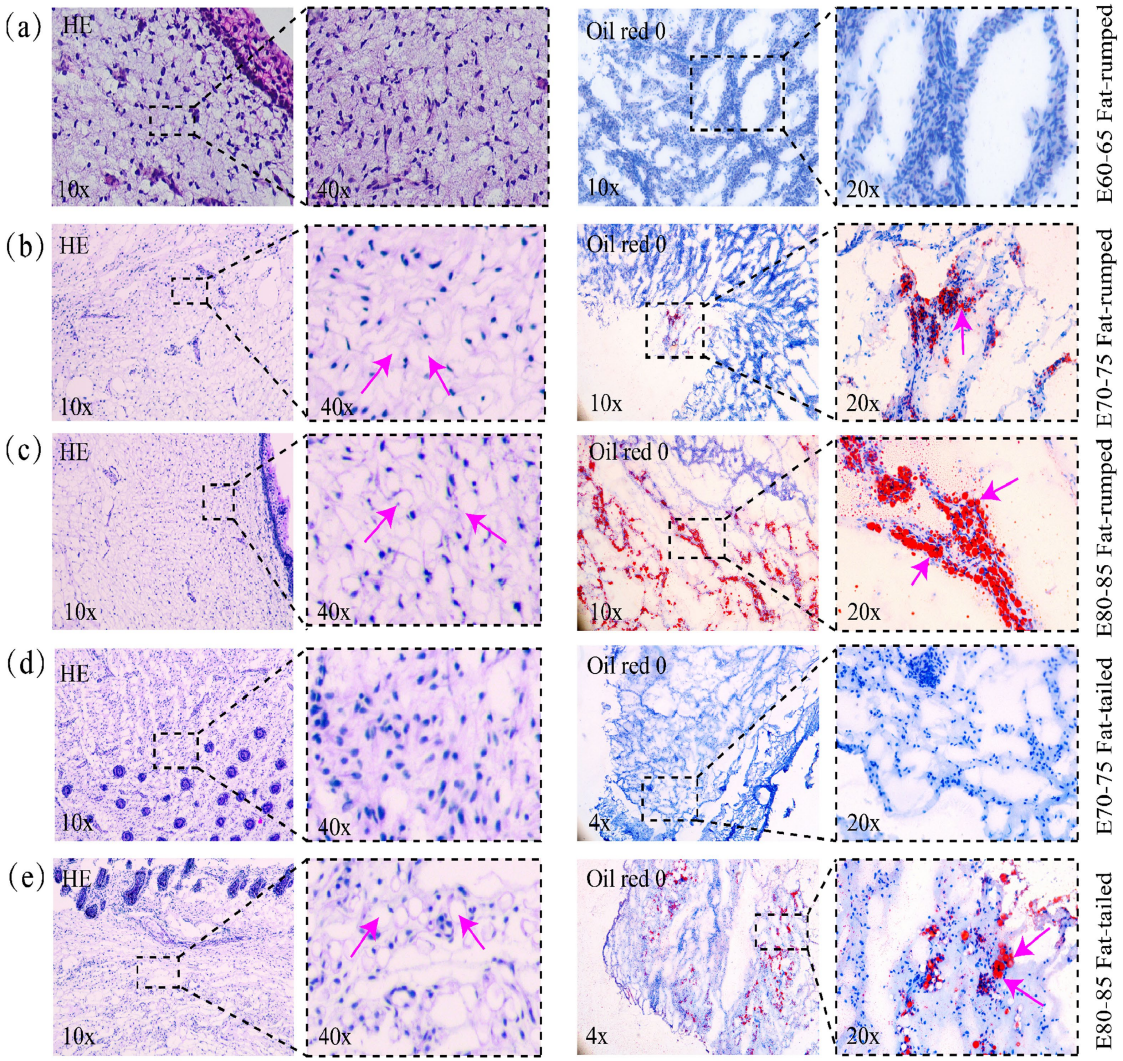
Sheep fetal frozen section by paraffin section HE staining, and Oil Red O staining. **(a)** E60–65 Fat-rumped. **(b)** E70–75 Fat-rumped. **(c)** E80–85 Fat-rumped. **(d)** E70–75 Fat-tailed. **(e)** E80–85 Fat-tailed. The lipid droplets turned orange red to bright red, and the nucleus was stained blue with hematoxylin.

To define the key developmental period for tail fat deposition, we analyzed fetuses of different tail types at various gestational ages using dissection, Oil Red O staining on frozen sections, and H&E staining on paraffin sections. Our analysis confirmed that visible tail fat formation and lipid droplet accumulation commence at approximately E80–85 days of gestation (corresponding to a fetal body length of 25 cm) (Figure 1; Supplementary Figure S1). Importantly, we identified an earlier, critical time point at E70-75 days of gestation (corresponding to a fetal body length of 16 cm), establishing this as the pivotal window for the onset of rump fat development in fat rumped sheep (Figure 1; Supplementary Figure S1).

Concomitantly, we profiled the expression of *PDGFD* and its receptors, *PDGFRα* and *PDGFRβ*, in embryonic tail and rump adipose tissues, we found that *PDGFD* expression exhibited a decreasing trend with increasing gestational age in both tail types (Supplementary Figure S2). We also observed that the expression level of *PDGFD* in the rump fat of fat-tailed sheep was higher than that in long-fat-tailed sheep. The expression pattern of the receptor *PDGFRα* was largely mirrored that of *PDGFD*. In contrast, *PDGFRβ* exhibited an opposing expression trend between the two tail types: it increased with gestational age in fat-rumped sheep but decreased in fat-tailed sheep (Supplementary Figure S2).

### 3.2 Isolation, culture, and characterization of ovine ADSCs

To mechanistically dissect PDGFD function, we established a physiologically relevant *in vitro* model. ADSCs were successfully isolated from embryonic sheep tail tissue and displayed characteristic fibroblast-like morphology (Supplementary Figure S3a–d). Molecular characterization confirmed expression of classic mesenchymal stem cell markers (CD44, CD90, CD29, CD106) and the absence of the endothelial marker CD31. Weak expression of the hematopoietic marker CD45 was also detected (Supplementary Figure S4).

**Figure 2:**
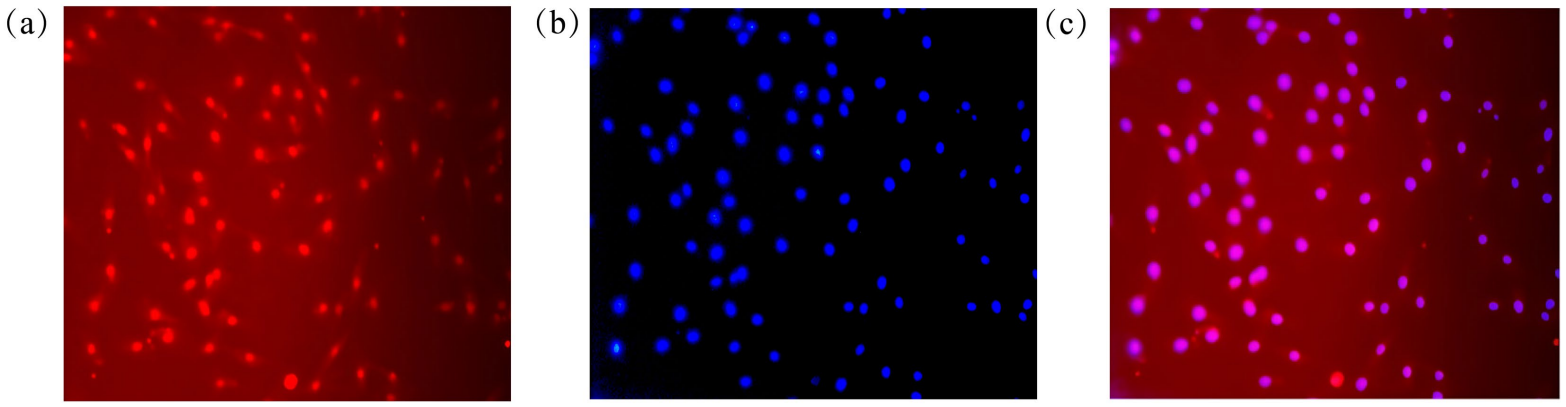
Immunofluorescence identification of CD44 in ovine ADSCs. **(a)** CD44 immunofluorescence staining (red) shows strong positive expression localized to the cell membrane and cytoplasm. **(b)** DAPI staining (blue) marks the cell nuclei. **(c)** Merged image illustrates the subcellular localization of CD44.

Furthermore, immunofluorescence analysis of the canonical MSC marker CD44 revealed strong positive staining that was localized to the cell membrane and cytoplasm, as expected. Counterstaining with DAPI confirmed nuclear integrity and cell density (Figure 2). Collectively, positive expression of CD44, CD106, CD29, and CD90, and the absence of CD31 and CD45 expression confirm the successful isolation and culture of a purified population of ovine ADSCs.

### 3.3 Osteogenic and Adipogenic differentiation potential of ADSCs

To assess their multilineage potential, fifth-passage ADSCs were subjected to osteogenic and adipogenic induction. Following osteogenic induction, ADSCs underwent a notable morphological shift, transitioning from a spindle-shaped to a more polygonal and cuboidal morphology, which was evident by day 3 and pronounced by day 6. Alizarin Red S staining on day 12 confirmed successful differentiation, revealing the presence of dense, dark red mineralized nodules, indicating extensive calcium deposition (Supplementary Figure S5). These results demonstrate the robust osteogenic differentiation capacity of the isolated ovine ADSCs.

Similarly, adipogenic induction also induced rapid morphological changes. Within 2 days, cells began to accumulate small, intracellular lipid droplets. By day 6, these droplets had coalesced into numerous large, spherical, and translucent droplets that filled the cytoplasm. Oil Red O staining on day 6 confirmed these structures to be neutral lipids, with bright red staining clearly visible (Supplementary Figure S6). This confirms the strong adipogenic potential of the ADSCs isolated from fetal sheep tail tissue.

### 3.4 Expression patterns of Adipogenic marker genes, *PDGFD*, and its receptors during differentiation

**Figure 3:**
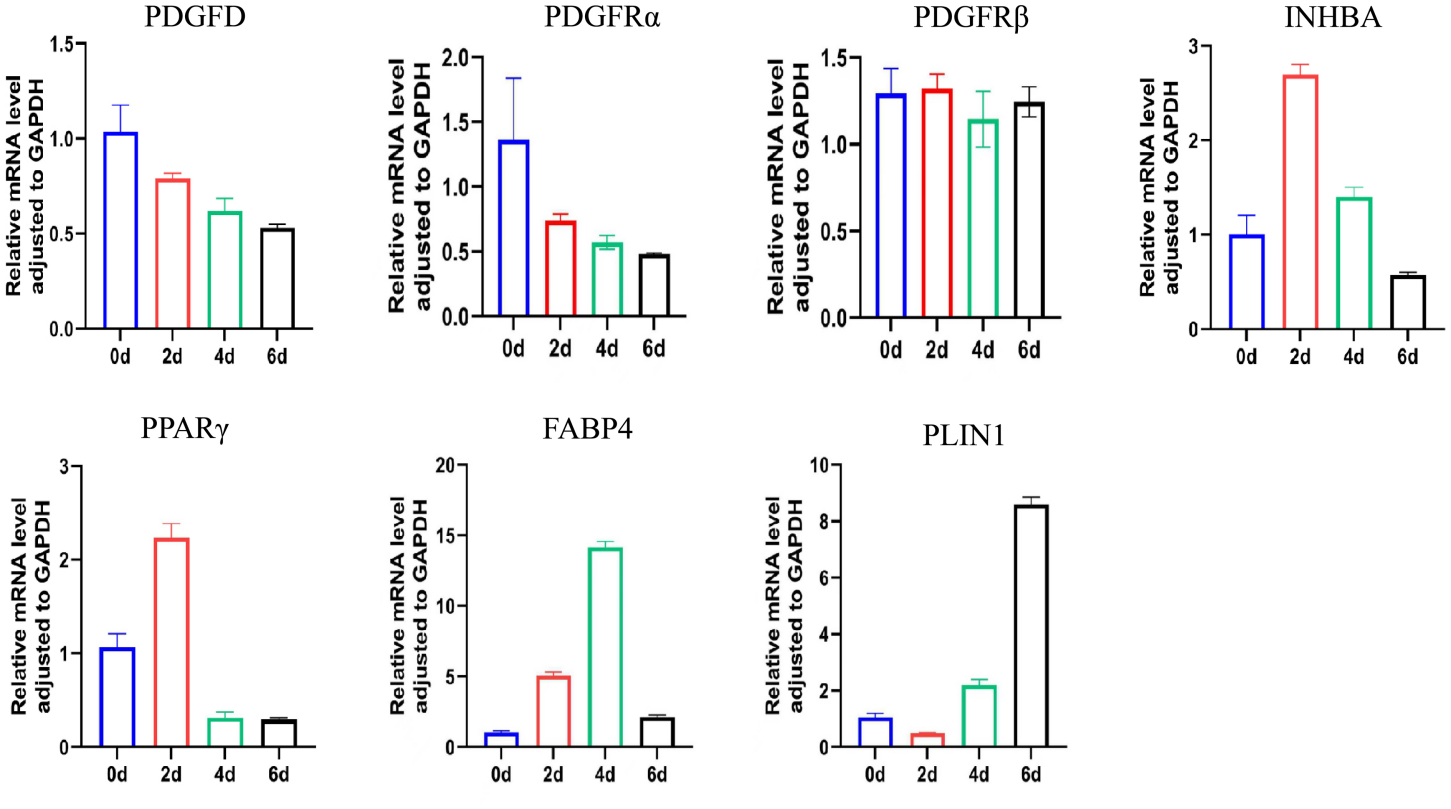
Expression of *PDGFD* and its receptor and key lipogenic genes.

To characterize the molecular events during adipogenesis, we profiled the expression of key adipogenic markers and the *PDGFD* signaling pathway in ADSCs undergoing differentiation (days 0, 2, 4, and 6). As expected, the expression of adipogenic genes was successfully induced, with each marker peaking at a distinct stage of differentiation (Figure 3). The master regulator PPARγ and the INHBA showed the highest expression at day 2. This was followed by a peak in the fatty acid-binding protein FABP4 at day 4, and finally, the lipid droplet-coating protein PLIN1 at day 6 (Figure 3).

Concurrently, we analyzed the expression of *PDGFD* and its receptors. A striking inverse correlation was observed: as adipogenic commitment progressed, the mRNA levels of both PDGFD and its receptor *PDGFRα* progressively decreased (Figure 3). In contrast, the expression level of *PDGFRβ* remained relatively stable throughout the differentiation process and did not show statistically significant variation (Figure 3).

#### 3.4.1 Efficient knockdown of *PDGFD* and its impact on cell morphology

**Figure 4:**
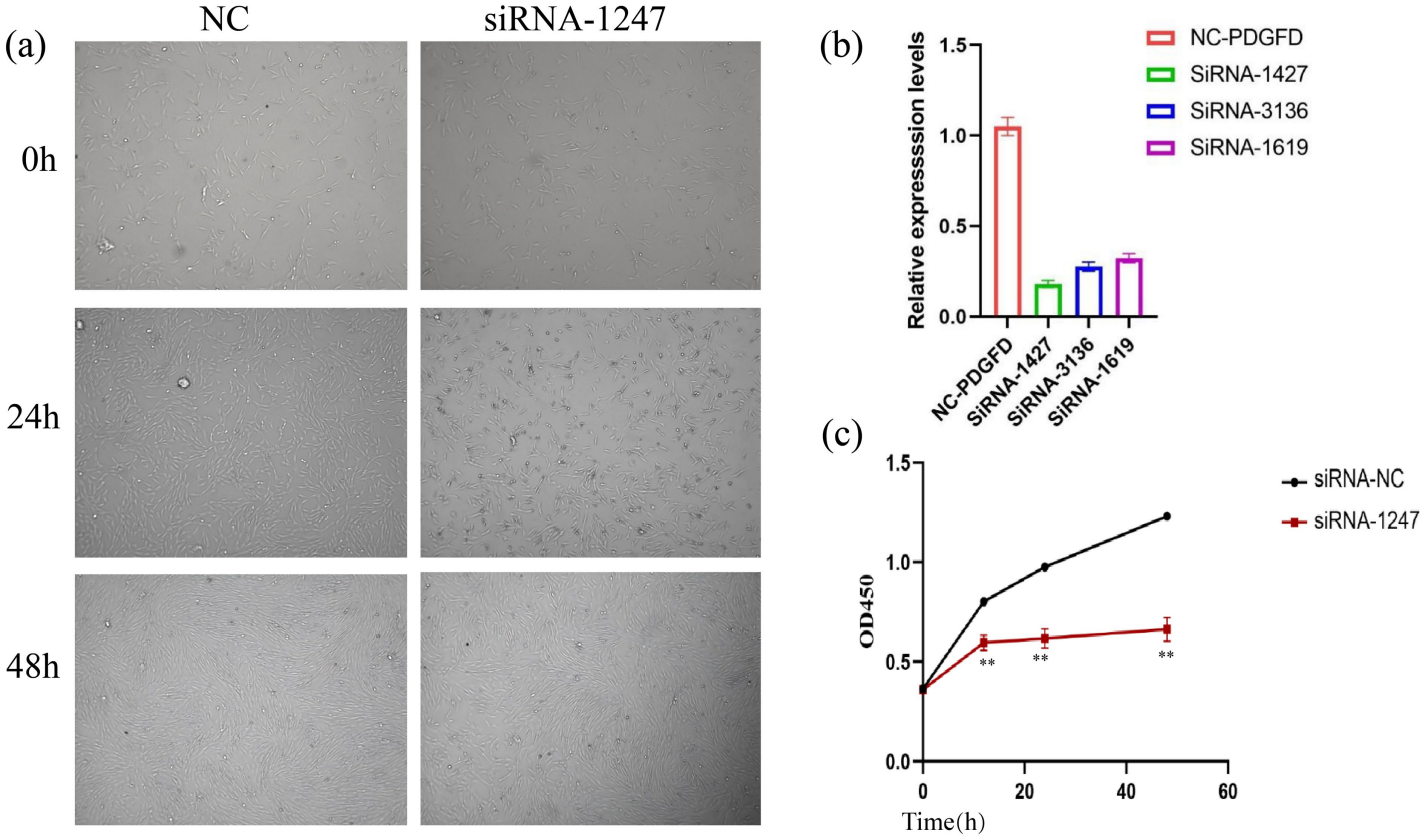
Knockdown of *PDGFD.* **(a)** ADSCs transfected with si-NC or si-PDGFD (si-1427) at 0, 24, and 48 h post-transfection. **(b)** Relative mRNA expression of *PDGFD* in ADSCs 24 h after transfection with three independent siRNAs (si-1619, si-1427, si-3136) or a negative control siRNA (NC). **(c)** Cell proliferation was assessed by CCK-8 assay over 48 h following transfection with si-NC or si-PDGFD (si-1427). Absorbance at 450 nm is proportional to cell viability. Data are presented as mean ± SD (*n* ≥ 3). **p ≤ 0.01.

To investigate the functional role of *PDGFD*, we transfected ovine ADSCs with three independent siRNAs targeting *PDGFD* (siRNA-1427, siRNA-3136, siRNA-1619) or a non-targeting negative control (siRNA-NC). RT-qPCR analysis at 24 h post-transfection confirmed highly efficient knockdown. The siRNA-1427 construct was the most effective, reducing *PDGFD* mRNA levels by 84% compared to the siRNA-NC group (*p* < 0.01) and was therefore selected for all subsequent functional experiments (Figure 4b).

Following the establishment of the knockdown model, we observed notable morphological changes. At 24 h post-transfection, the si-PDGFD group exhibited a significantly lower cell density and an increase in rounded, non-viable cells compared to the control, suggesting potential cytotoxicity or impaired proliferation (Figure 4a). However, 48 h after the initial observation, the remaining cells appeared healthy and resumed a normal morphological appearance (Figure 4a).

#### 3.4.2 *PDGFD* knockdown impairs ADSC proliferation

To quantitatively assess the impact on proliferation, we performed a CCK-8 assay following si-*PDGFD* transfection. The results demonstrated that *PDGFD* knockdown significantly suppressed the proliferative capacity of ADSCs over a 48-h period compared to the siRNA-NC control group (Figure 4c).

### 3.5 Transcriptomic profiling reveals *PDGFD* regulates signaling pathways in ADSCs

**Figure 5:**
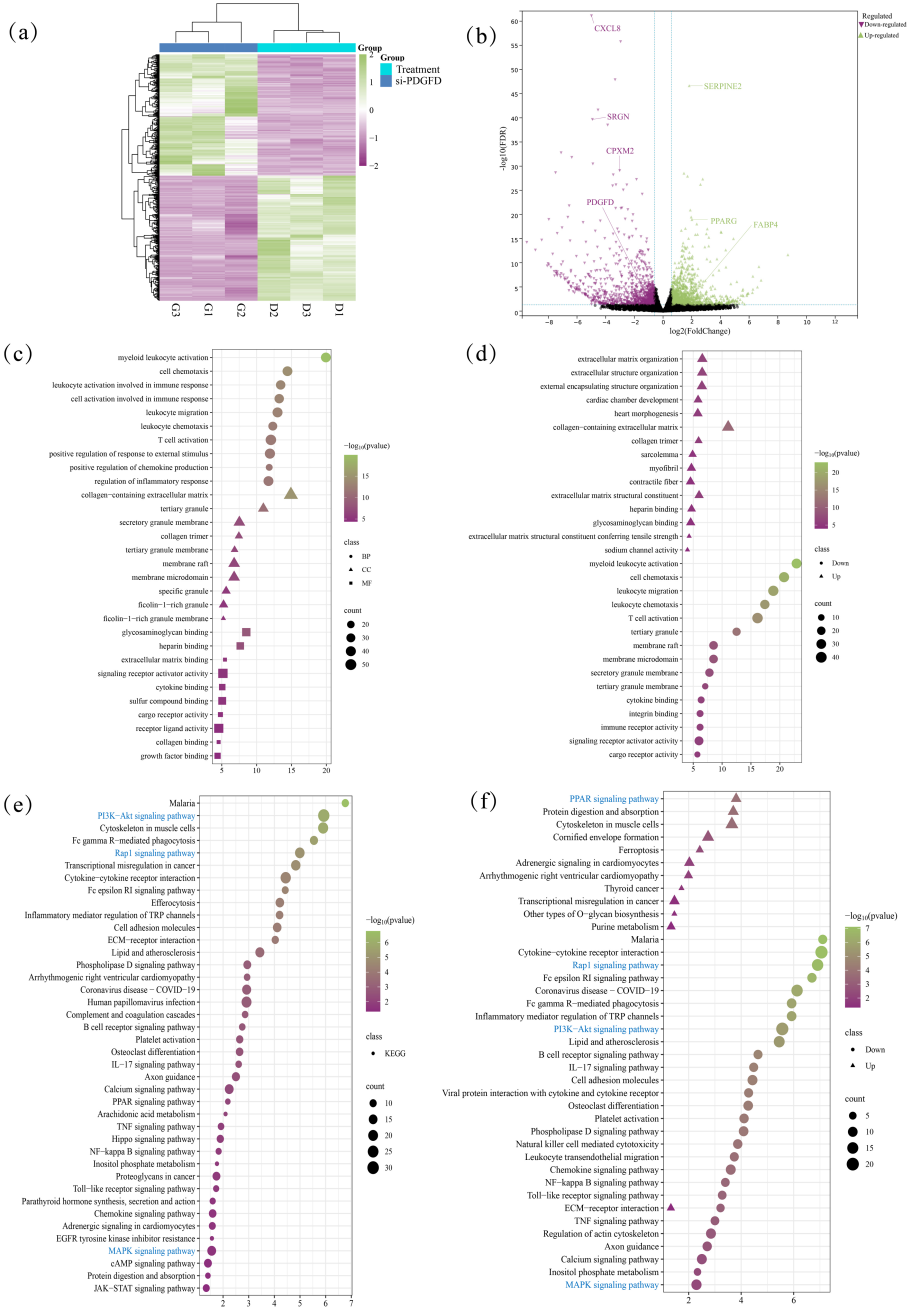
Differential gene analysis after 48 h of *PDGFD* interference: **(a)** Differential gene clustering heat map; **(b)** Differential gene volcano map; **(c)** differential gene GO enrichment analysis; **(d)** up-down-regulated gene GO enrichment analysis; **(e)** differential gene KEGG enrichment analysis; **(f)** up-down-regulated gene KEGG enrichment analysis.

To elucidate the global transcriptional changes and transcriptional network regulated by *PDGFD*, we performed RNA-seq on ADSCs 48 h after transfection with si-PDGFD and si-NC. Knockdown of *PDGFD* resulted in 915 differentially expressed genes (DEGs) (*p* ≤ 0.05, |log_2_FoldChange| ≥ 1), comprising 451 upregulated and 464 downregulated genes (Figure 5a; Supplementary Table S4). Notably, the most significantly altered genes included the profoundly downregulated chemokine *CXCL8* and the upregulated serine protease inhibitor *SERPINE2*, and the adipogenic markers *PPARγ* and *FABP4* were also significantly upregulated (Figure 5b).

Gene Ontology (GO) enrichment analysis of all DEGs revealed a strong association with biological processes including cell adhesion, inflammatory response, and MAPK cascade (Figure 5; Supplementary Table S4). When analyzed separately, the downregulated genes were predominantly linked to inflammatory response, cell adhesion, and MAPK cascade, while upregulated genes were enriched in processes such as transcription and phosphorylation (Figure 5d; Supplementary Tables S6, S7).

Kyoto Encyclopedia of Genes and Genomes (KEGG) pathway analysis further demonstrated that *PDGFD* knockdown significantly impacted pathways related to signal transduction and the immune system (Figure 5e). Crucially, this analysis confirmed the enrichment of the PI3K-Akt and MAPK signaling pathways, established downstream cascades of *PDGF* signaling. A focused analysis showed that upregulated genes were involved in metabolic pathways like the *PPAR* signaling pathway, whereas downregulated genes were strikingly overrepresented in immune and signaling pathways, including the PI3K-Akt signaling pathway, Rap1 signaling pathway, and Toll-like receptor signaling pathway (Figure 5f).

To further capture more subtle and coordinated expression changes across the entire transcriptome, we performed Gene Set Enrichment Analysis (GSEA) on all genes. This analysis corroborated and extended the findings above, showing a significant positive enrichment (normalized enrichment score, NES = −1.17, *p <* 0.05) for the MAPK signaling pathway, alongside significant enrichment of metabolic pathways such as PPAR signaling, fatty acid metabolism, and tyrosine metabolism (Supplementary Figure S10; Supplementary Table S8). Notably, the GSEA results specifically highlighted the enrichment of *PDGFD*’s cognate receptor, *PDGFRβ*, within the MAPK signaling pathway gene set, providing direct bioinformatic evidence linking PDGFD knockdown to the perturbation of this key downstream cascade. This independent, global analysis underscores the central role of PDGFD in regulating both key signaling and metabolic programs in ADSCs.

### 3.6 RT-PCR validation of transcriptomic sequencing results

**Figure 6:**
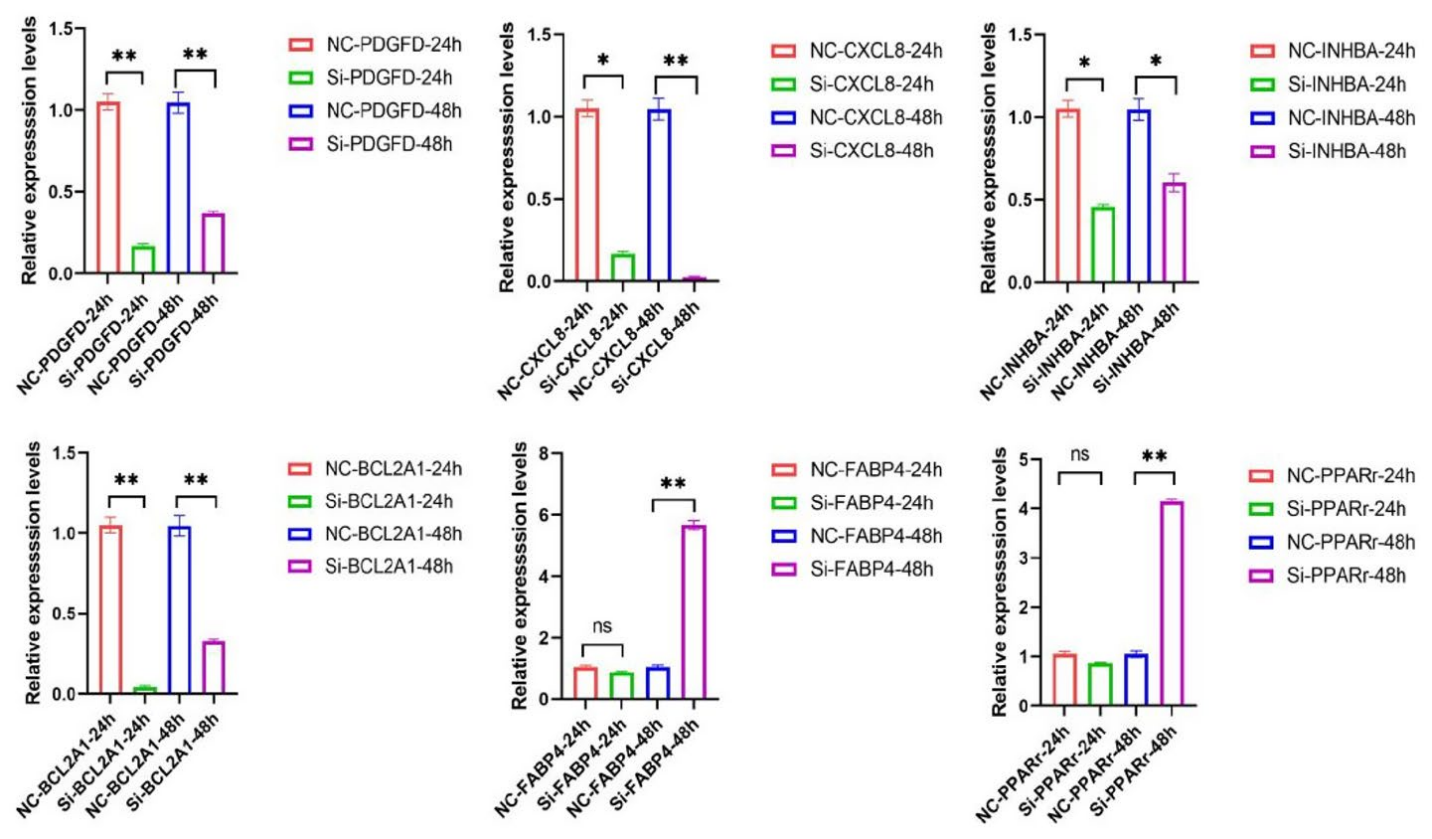
RT-qPCR verification after 24 h and 48 h of interference. *p ≤ 0.05. **p ≤ 0.01.

To confirm the reliability of our transcriptomic data, we performed RT-PCR on a panel of key genes using the same RNA samples submitted for sequencing. We also included an earlier time point (24 h) to track initial transcriptional changes. The expression trends for all tested genes at 48 h were fully consistent with the RNA-seq data (Figure 6). This included the significant upregulation of the adipogenic markers *PPARγ* and *FABP4*, and the pronounced downregulation of *CXCL8*, *INHBA*, *BCL2A1* and *PDGFD* itself.

### 3.7 *PDGFD* knockdown alters key signaling pathways

To visualize the impact of *PDGFD* knockdown on specific signaling cascades, we constructed schematic diagrams of the PI3K-Akt and MAPK pathways based on our KEGG enrichment results, mapping the differentially expressed genes (DEGs) onto their respective pathway positions (Supplementary Figures S7, S8). In the PI3K-Akt signaling pathway, a predominant downregulation of genes was observed. Key affected genes included several phosphatidylinositol kinases such as *PIK3CG*, *PIK3CD*, *PIK3R1*, *PIK3R5*, and *PIK3AP1* (Supplementary Figure S7). The MAPK signaling pathways exhibited a more mixed regulatory pattern. Notably, genes involved in the ERK cascade like ERK1 and MAPK8IP1were upregulated. In contrast, several genes associated with the JNK cascade (*MAP3K13*, *MAP3K5*, and *MAPK8*) were downregulated (Supplementary Figure S8).

### 3.8 Protein–protein interaction network implicates *CXCL8* in *PDGFD* signaling

Given its status as the most significantly downregulated gene and its presence in the enriched KEGG pathways, we investigated the connectivity of *CXCL8*. A protein–protein interaction (PPI) network was generated using the STRING database, incorporating *CXCL8* and the DEGs from the PI3K-Akt and MAPK signaling pathways. The resulting network revealed a high-confidence (interaction score > 0.7) and interconnected web of proteins (Supplementary Figure S9). CXCL8 was a central node within this network, demonstrating multiple direct and indirect interactions with other downstream effectors, including components of both the PI3K-Akt and MAPK pathways.

## 4 Discussion

The fat tail in sheep is a specialized organ that has evolved for energy storage and metabolism, providing a critical adaptation to extreme environments. This adaptive capacity is underpinned by the pronounced plasticity of tail adipose tissue. Previous genomic studies have consistently identified *PDGFD* as a key candidate gene underlying the fat-tail phenotype, with evidence suggesting it promotes preadipocyte proliferation while inhibiting differentiation (10, 13). In this study, we built upon this genetic evidence by establishing a functional cellular model using ADSCs from tail adipose tissue in fetal sheep. Our results demonstrate that knockdown of *PDGFD* significantly attenuates ADSCs proliferation, providing direct *in vitro* validation of its pro-proliferative role. Furthermore, our transcriptomic analysis uncovered a profound downregulation of the chemokine gene *CXCL8* following *PDGFD* knockdown. Concurrently, we observed a significant upregulation of the key adipogenic markers *PPARγ* and *FABP4*, indicating a shift in cellular fate from proliferation towards differentiation. Functional enrichment and protein–protein interaction analyses suggest a model whereby the PDGFD-CXCL axis exerts its effects through the PI3K-Akt and MAPK signaling pathways, thus point to a complex regulatory network controlling ADSC fate. Collectively, our findings reveal a crucial role for *PDGFD*, potentially mediated by *CXCL8* and downstream signaling cascades, in modulating the proliferative and differentiation potential of ovine ADSCs, which is a key determinant of adipose tissue plasticity.

As an important organ in sheep, the tail adipose tissues can change in size dramatically in response to an energy surplus or deficit and are highly plastic and dynamic, and plays a critical role in their ability to survive and thrive in extreme environments (7). However, the developmental pried for fat tail formation is limited. We identified a fetal body length of 16 cm (E70) as the critical window for the initial formation of rump fat in Kazakh sheep. Whereas tail adipose tissue formation in fat-tailed sheep varieties concentrates around E80 (crown-rump length 25 cm). These findings align with previous studies indicating that E80 marks the key developmental stage for tail fat deposition in Tan fetal lambs (10). Specifically, at E80, preadipocytes in the tail region of Tan fetal lambs undergo extensive proliferation and differentiation into adipocytes, initiating rapid tail fat accumulation.

To functionally dissect *PDGFD*’s role, we established a knockdown model in ovine ADSCs. Our data provide a clear resolution to existing contradictions in the literature. We demonstrate that interference *PDGFD* significantly attenuates ADSC proliferation and concurrently initiates a robust differentiation program, as evidenced by the marked upregulation of the master regulators *PPARγ* and *FABP4*, as reported, *PPARγ* is an essential regulator of adipogenesis (27), *FABP4* is essential for cellular lipid regulation, membrane-protein interactions, and the modulation of metabolic and inflammatory pathways (28). Increased expression of *PPARγ* and *FABP4* after transfection with si-*PDGFD* can therefore directly regulate and modulate fat synthesis and deposition within cell. This mechanism enables the tail-rump adipose tissue to respond to nutritional and environmental stresses through its heterogeneity, enhancing sheep adaptability in extreme environments. This supports the model that *PDGFD* acts to maintain a proliferative, undifferentiated progenitor state (13). However, Li suggested that *PDGFD* could inhibited proliferation but enhanced differentiation, the *PDGFD* gene expression was consistently negatively correlated with fat deposition in sheep tails (9). Thus, reflecting model-specific differences or the complex context-dependency of *PDGF* signaling. Furthermore, the downregulation of the anti-apoptotic gene *BCL2A1* following *PDGFD* knockdown reveals an additional mechanism-enhanced progenitor survival-through which *PDGFD* secures a large cellular reservoir for future fat deposition.

Platelet-derived growth factor has been directly implicated in developmental and physiological processes (29) and regulate energy metabolism in obesity (30). *PDGFRβ* signaling has an essential role in inhibiting differentiation of white adipocytes by regulating the expression of *PPARγ* and C/ *EBPα*, which were identified as the key transcriptional regulators of adipogenesis (31). In our study, the concurrent decrease in *PDGFD* and *PDGFRα* expression we observed throughout gestation and during *in vitro* differentiation strongly suggests that this pathway is most active during these early phases, functioning to build the cellular foundation of the adipose depot rather than to execute terminal differentiation. This is consistent with the conclusions of Dong et al. (10) regarding the initial formation of tail adipose tissue in Tan fetal lambs. This also aligns with previous reports of higher *PDGFD* expression in fat-tailed sheep compared to thin-tailed breeds, suggesting that elevated *PDGFD* levels may facilitate excessive fat deposition by maintaining a larger pool of adipose progenitor cells (10). Our results reveal that *PDGFD* play important regulatory roles in promoting angiogenesis and inhibiting the differentiation of ADSCs in the tail adipose tissues of fetal lambs.

Research has demonstrated that *PDGFD* participates in multiple signaling pathways, including the activation of PI3K and MAPK pathways (32). Our transcriptomic analysis yielded a pivotal mechanistic insight: the profound downregulation of the chemokine *CXCL8* emerged as the most significant consequence of *PDGFD* knockdown. This positions CXCL8 as a critical downstream effector. The known biology of *CXCL8* - a regulator of proliferation, angiogenesis, and a player in obesity (33) that activates PI3K/Akt and MAPK pathways-makes it a biologically plausible mediator. This connection was reinforced by our protein–protein interaction network, which placed *CXCL8* as a central node within the *PDGFD* regulatory network (34–38). We, therefore, propose a coherent model: during early development, *PDGFD* signaling sustains *CXCL8* expression. This PDGFD-CXCL8 axis, in turn, maintains the activity of key pro-proliferative and pro-survival pathways. Our data refine this further, showing that *PDGFD* knockdown leads to upregulation of the ERK cascade and downregulation of the JNK cascade, suggesting a precise re-routing of MAPK signaling toward a pro-growth, anti-apoptotic state. The collapse of this circuit upon *PDGFD* knockdown halts proliferation and removes a brake on differentiation, enabling the adipogenic program to proceed. These findings significantly advance our understanding of the developmental biology of tail adipose tissue and highlight the *PDGF* signaling pathway as a critical regulator of tail adipose tissue plasticity. However, how PDGFD-CXCL8 axis regulate signaling pathways PI3K-Akt and MAPK to determine tail adipose tissue plasticity in the sheep remains elusive.

*PDGFD* is highly expressed by ADSCs, where it acts as a potent mitogenic factor. *PDGFD* also upregulates growth factor expression in ASCs (14). The *PDGFD* gene can upregulate the expression of *PPARγ* and *FABP4* in adipose-derived stem cells. Through the PI3K/Akt pathway, it regulates the proliferation and migration of adipose-derived stem cells, while upregulation of *PDGFD* increases the phosphorylation of Akt and ERK1/2 molecules in these cells, thereby affecting the proliferation and migration of preadipocytes (39). However, further research is warranted to ascertain these findings. In summary, *PDGFD* is highly expressed by ADSCs, where it acts as a potent mitogenic factor.

## 5 Conclusion

In conclusion, our study defines the initial formation of ovine rump and tail adipose tissue at E70-E80 as a critical developmental window. We demonstrate that *PDGFD* maintains ADSCs in a proliferative state while actively suppressing their differentiation into adipocytes. Mechanistically, we identified the chemokine *CXCL8* as a crucial downstream effector, forming a PDGFD-CXCL8 axis that co-regulates this process through the PI3K-Akt and MAPK/ERK signaling pathways. Collectively, our findings establish a model wherein high *PDGFD* expression during development expands the progenitor cell pool via *CXCL8*, creating the cellular foundation for the massive fat deposition characteristic of fat-tailed sheep. This provides a fundamental mechanistic explanation for how this gene, selected during domestication, contributes to an adaptive trait. The outstanding question of how environmental and nutritional signals modulate this PDGFD-CXCL8 axis *in vivo* to dynamically remodel adipose tissue presents a compelling direction for future research.

## Data Availability

The data presented in this study are deposited on the GSA database (accession: CRA037546), https://ngdc.cncb.ac.cn/gsa/browse/CRA037546

## Footnotes

1
https://string-db.org
