# Supplementary material for: PDGFD maintains ovine tail ADSCs in a proliferative state by suppressing CXCL8 and activating PI3K/MAPK signaling
Source: Front Vet Sci. 2026 Feb 13;13:1777426. doi: 10.3389/fvets.2026.1777426 (PMC12947266; doi:10.3389/fvets.2026.1777426)
Supplement: Supplementary file 1 [file Data_Sheet_1.docx]

Supplementary Material

# Supplementary Figures
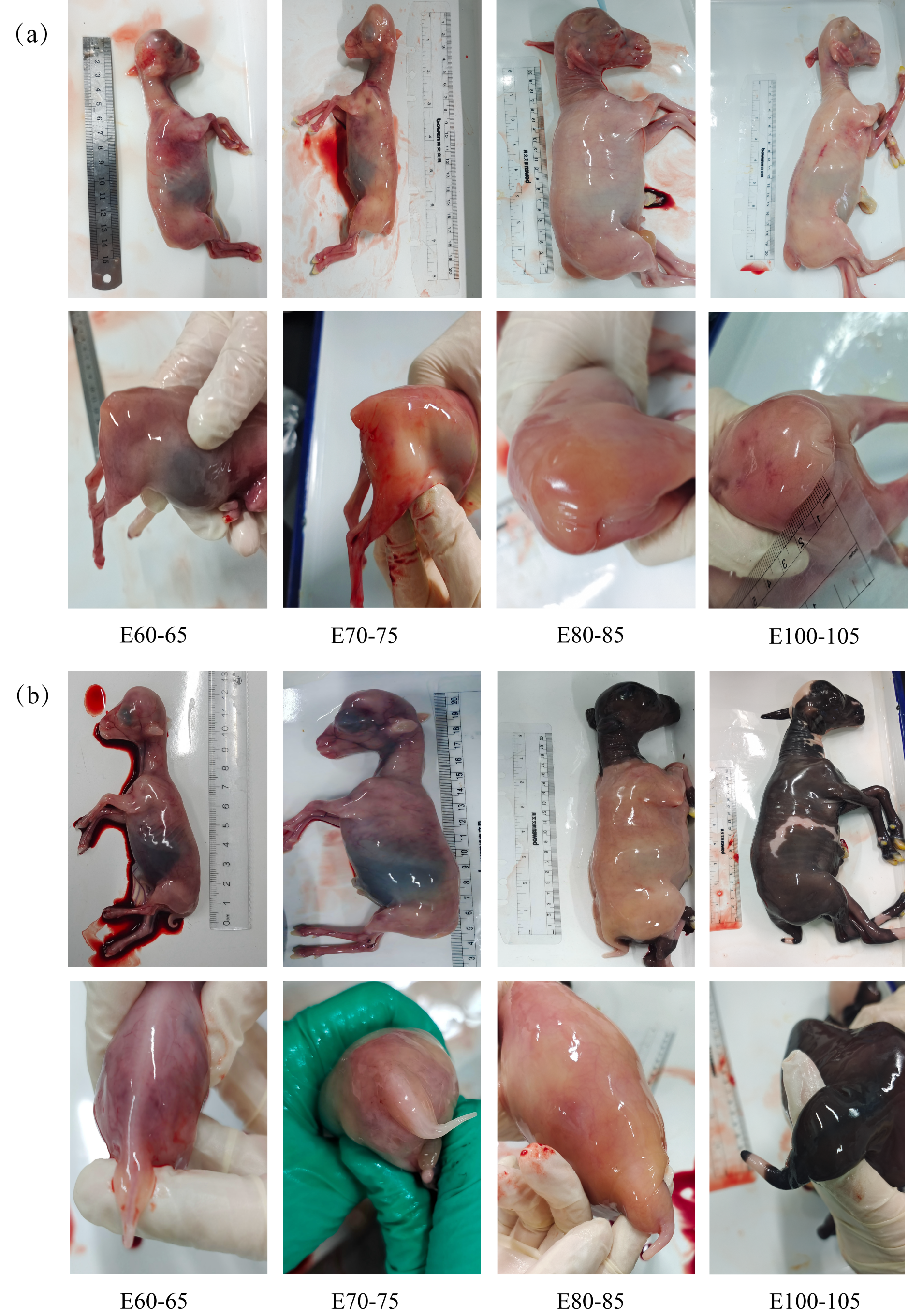


**Supplementary Figure 1.** Sheep fetal and their tail types: (a) Fat-rumped fetal sheep; (b) Fat-tailed fetal sheep.


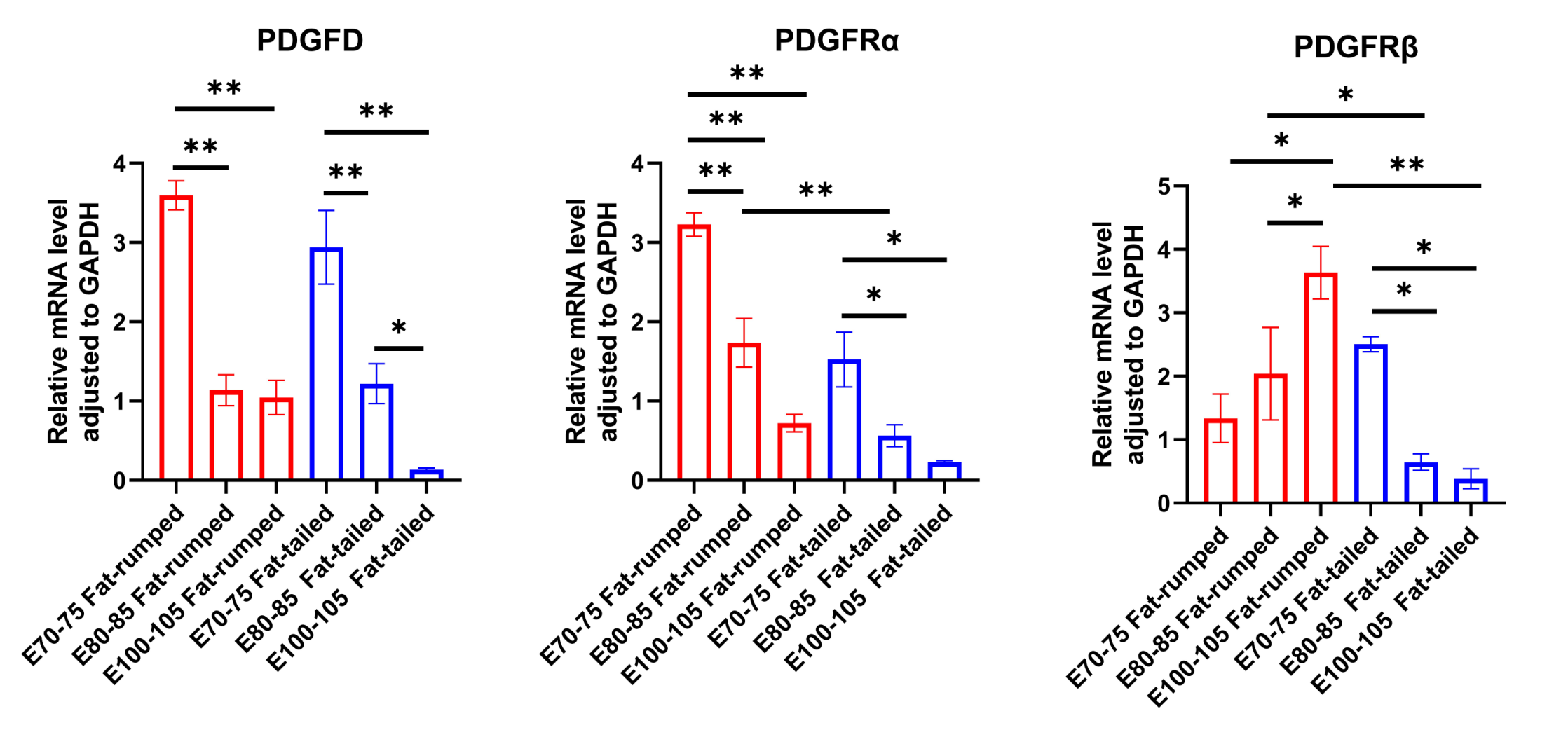


**Supplementary Figure 2.** Expression of PDGFD and its receptor in tail tissues of different tail types and developmental fetuses. Note: *:（*p*<0.05）, **:（*p*<0.01）.


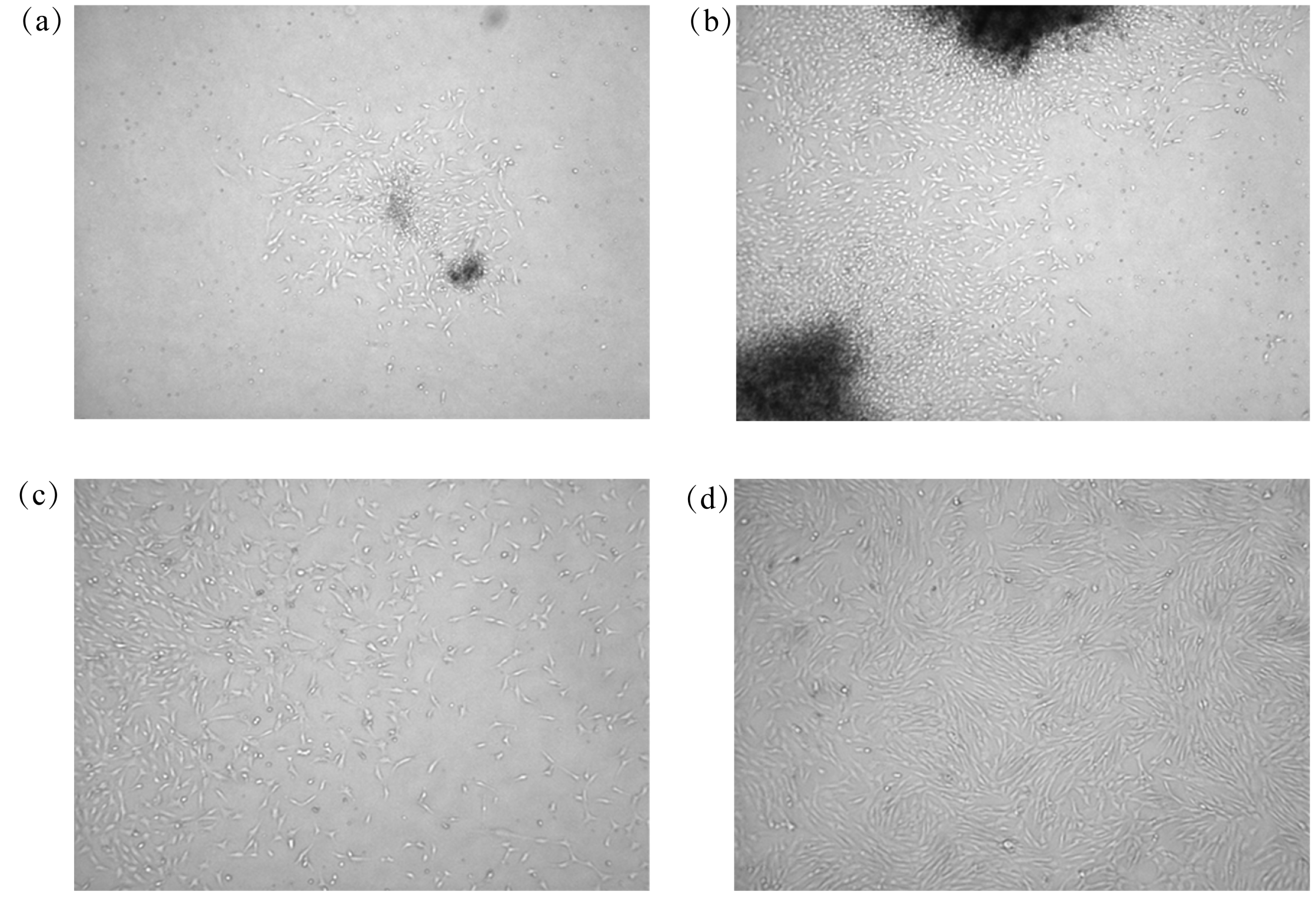


**Supplementary Figure 3.** Growth of primary adipose mesenchymal stem cells in sheep fetal sheep: (a) Day 1 of primary culture; (b) Day 3 of primary culture; (c) Day 7 of primary culture; (d) Transfer to 5th generation cells.


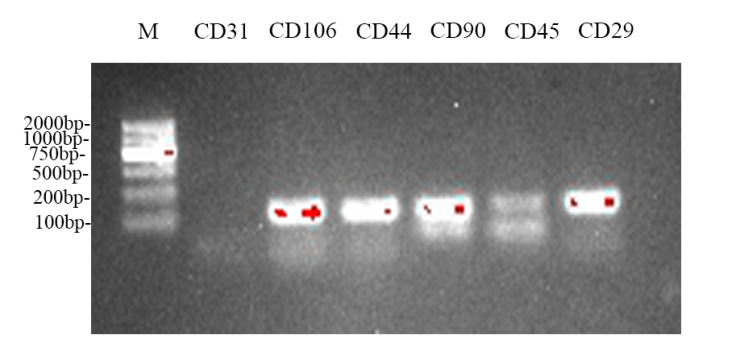


**Supplementary Figure 4.** Adipose mesenchymal stem cell marker agarose gel electrophoresis.


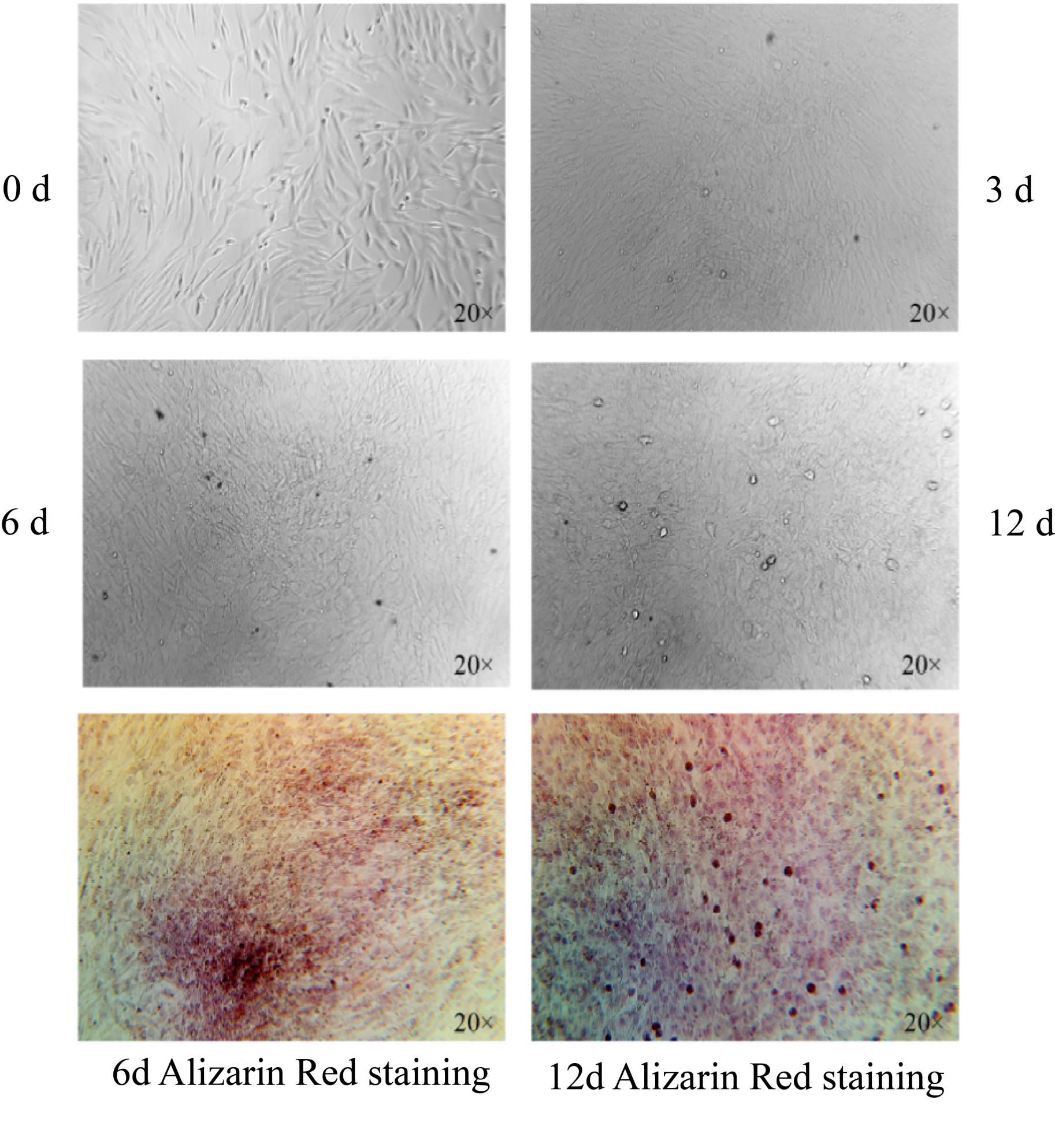


**Supplementary Figure 5.** Osteogenic differentiation of adipose mesenchymal stem cells.


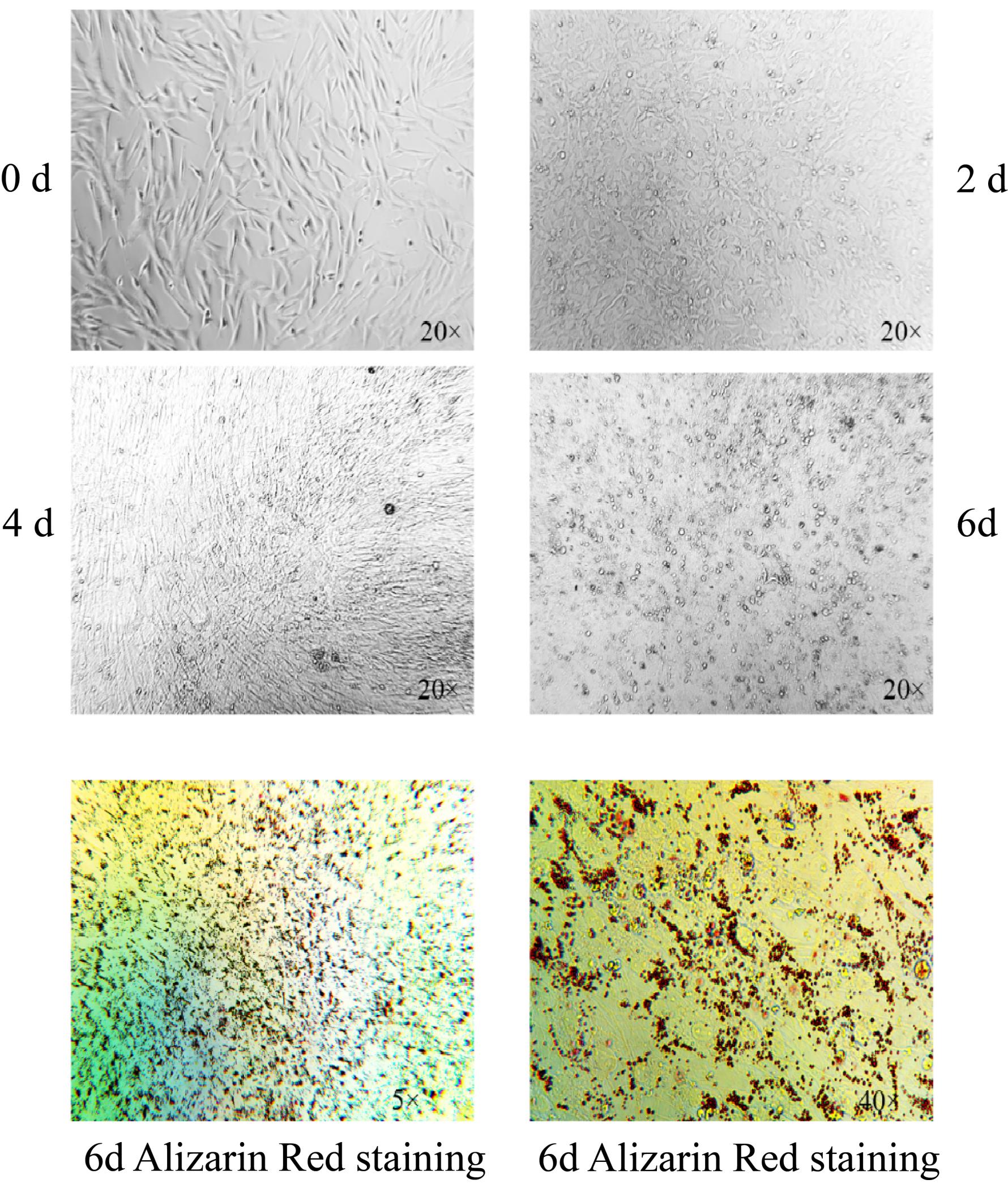


**Supplementary Figure 6.** Adipose mesenchymal stem cells differentiate into lipids.


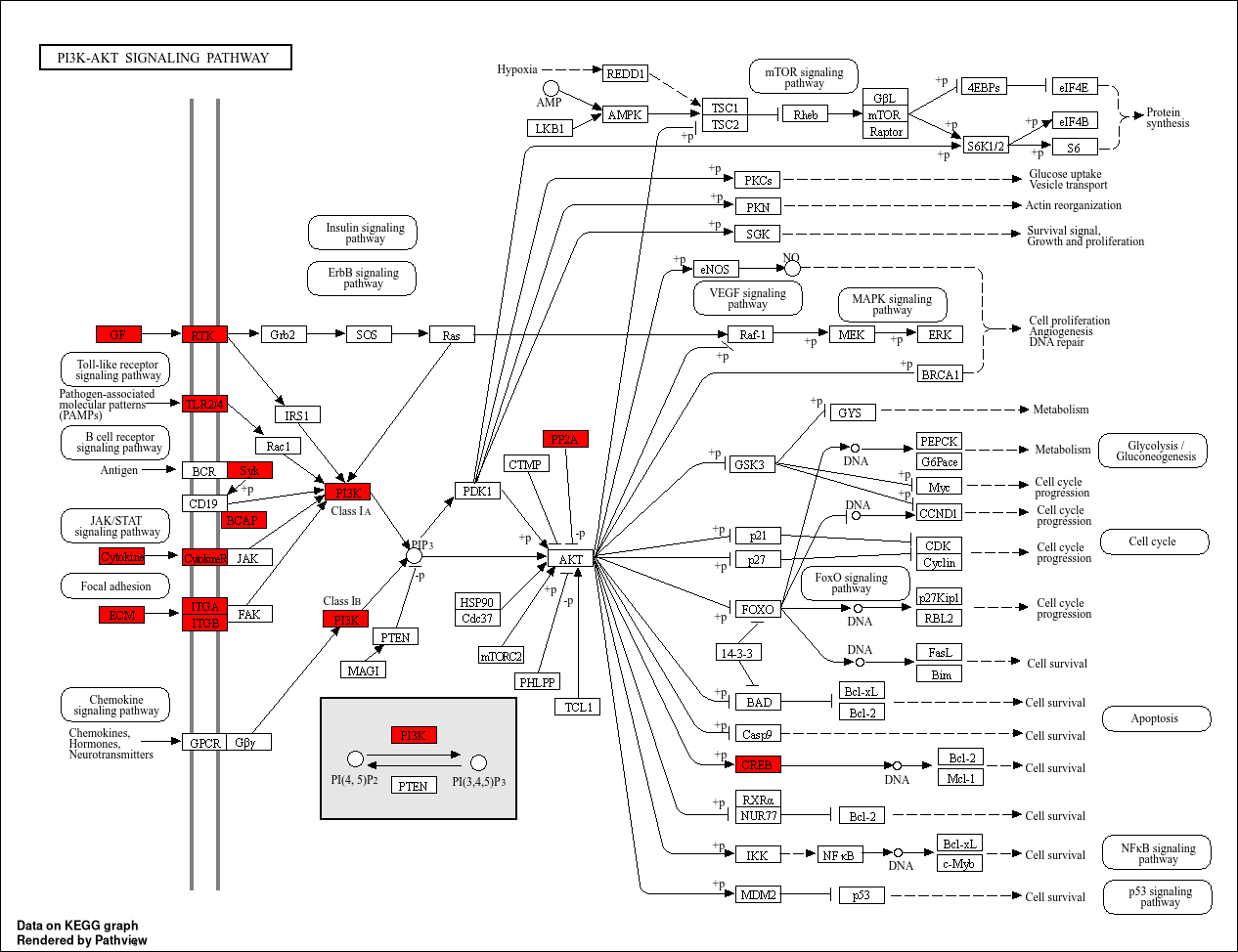


**Supplementary Figure 7.** PI3K-Akt signaling pathway regulation diagram.© Kanehisa Laboratories.


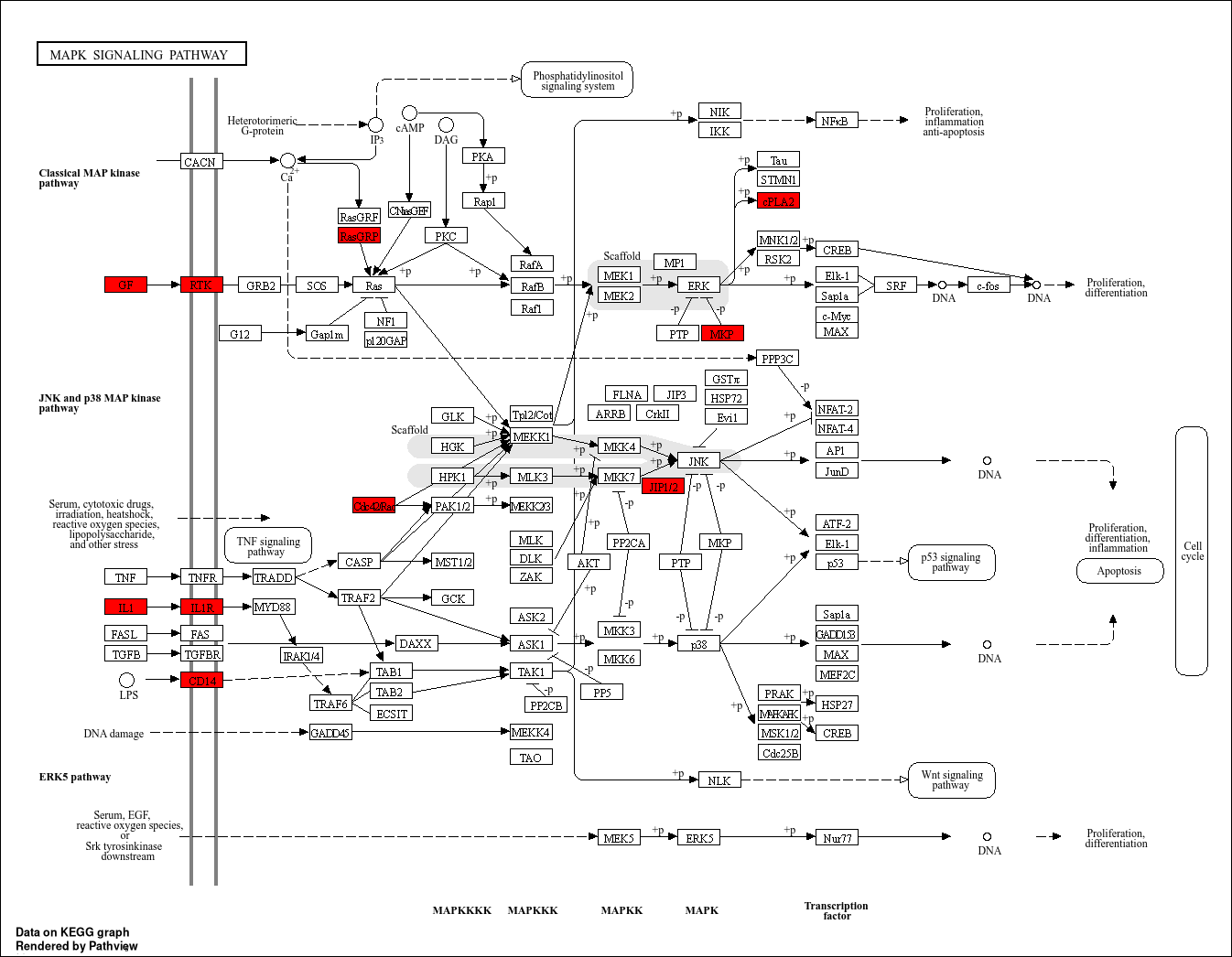


**Supplementary Figure 8.** MAPK signaling pathway regulation diagram.© Kanehisa Laboratories.


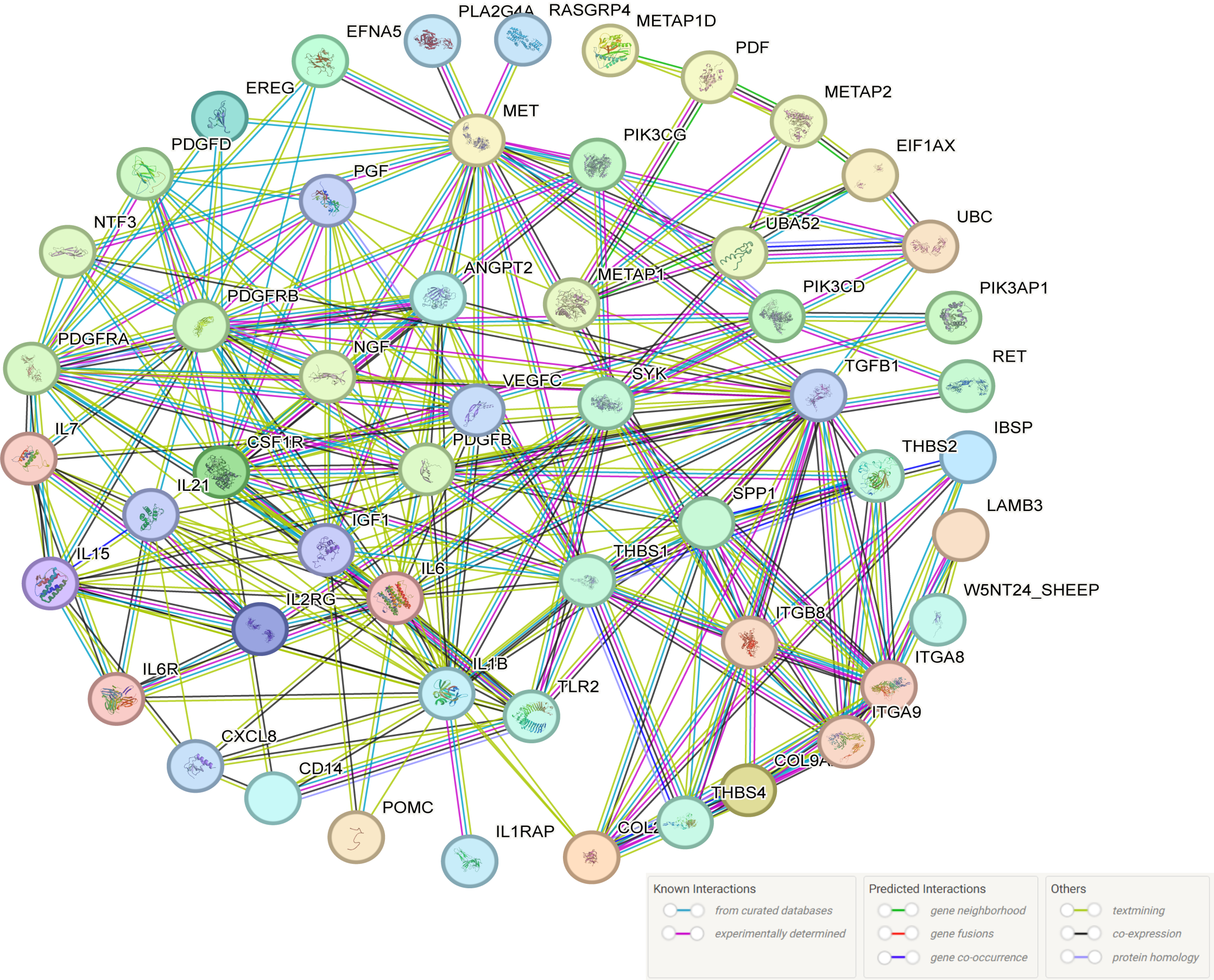


**Supplementary Figure 9.** Protein interaction analysis network diagram.


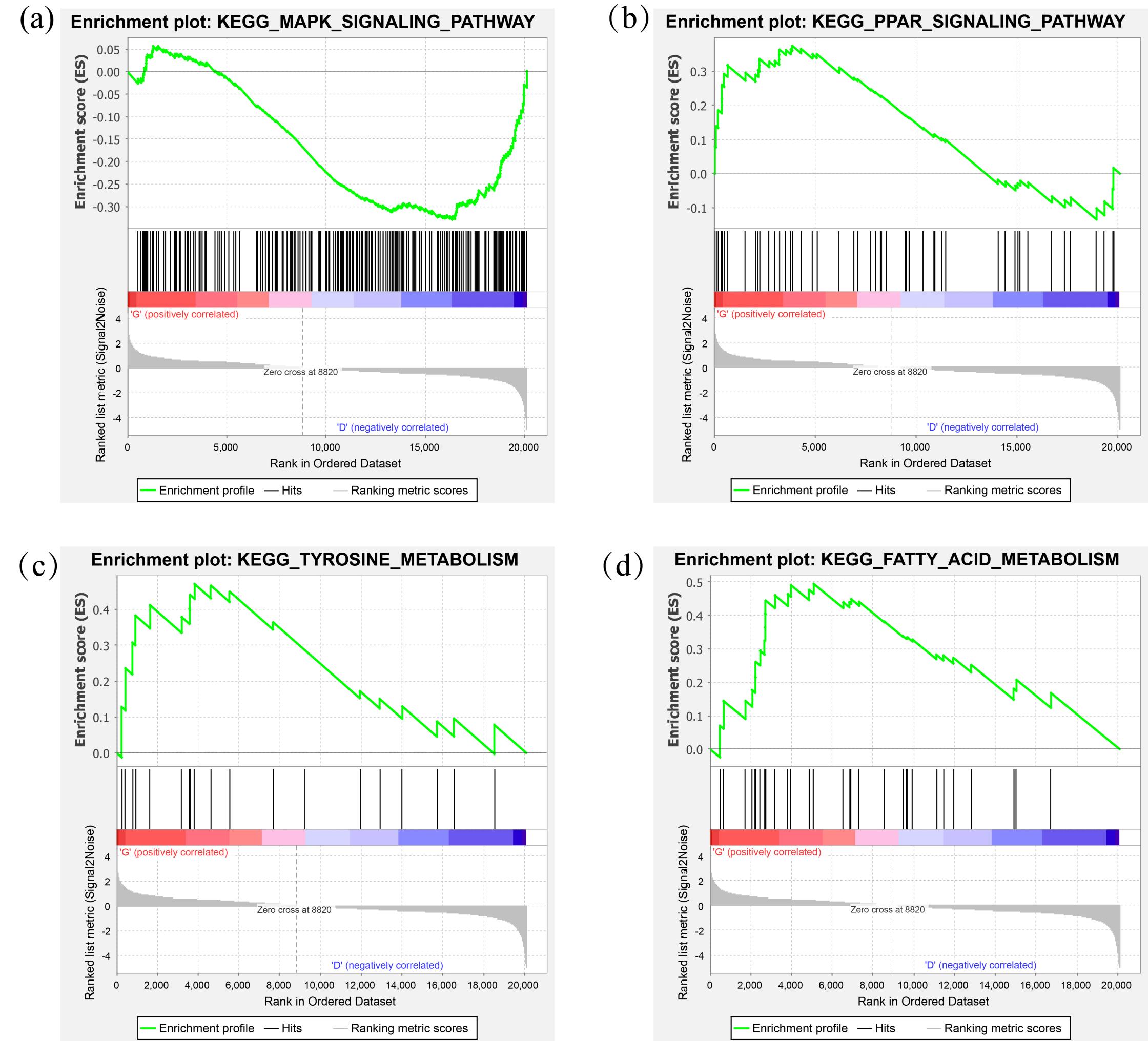


**Supplementary Figure 10.** GSEA enrichment assay pathway.
